# Transformation and Articulation of Clinical Data to Understand Students’ and Health Professionals’ Clinical Reasoning: Protocol for a Scoping Review

**DOI:** 10.2196/50797

**Published:** 2023-12-13

**Authors:** Marie-France Deschênes, Nicolas Fernandez, Kathleen Lechasseur, Marie-Ève Caty, Dina Azimzadeh, Tue-Chieu Mai, Patrick Lavoie

**Affiliations:** 1 Faculté des sciences infirmières Université de Montréal Montréal, QC Canada; 2 Faculté de Médecine Université de Montréal Montréal, QC Canada; 3 Faculté des sciences infirmières Université Laval Québec, QC Canada; 4 Département d'orthophonie Université du Québec à Trois-Rivières Trois-Rivières, QC Canada; 5 Faculté des sciences infirmières Université de Montréal Montreal, QC, QC Canada

**Keywords:** clinical reasoning, semantic qualifiers, discourse, linguistics, education, natural language processing, scoping review, clinical data, educational strategy, student, health care professional, semantic transformation

## Abstract

**Background:**

There are still unanswered questions regarding effective educational strategies to promote the transformation and articulation of clinical data while teaching and learning clinical reasoning. Additionally, understanding how this process can be analyzed and assessed is crucial, particularly considering the rapid growth of natural language processing in artificial intelligence.

**Objective:**

The aim of this study is to map educational strategies to promote the transformation and articulation of clinical data among students and health care professionals and to explore the methods used to assess these individuals’ transformation and articulation of clinical data.

**Methods:**

This scoping review follows the Joanna Briggs Institute framework for scoping reviews and the PRISMA-ScR (Preferred Reporting Items for Systematic Reviews and Meta-Analyses Extension for Scoping Reviews) checklist for the analysis. A literature search was performed in November 2022 using 5 databases: CINAHL (EBSCOhost), MEDLINE (Ovid), Embase (Ovid), PsycINFO (Ovid), and Web of Science (Clarivate). The protocol was registered on the Open Science Framework in November 2023. The scoping review will follow the 9-step framework proposed by Peters and colleagues of the Joanna Briggs Institute. A data extraction form has been developed using key themes from the research questions.

**Results:**

After removing duplicates, the initial search yielded 6656 results, and study selection is underway. The extracted data will be qualitatively analyzed and presented in a diagrammatic or tabular form alongside a narrative summary. The review will be completed by February 2024.

**Conclusions:**

By synthesizing the evidence on semantic transformation and articulation of clinical data during clinical reasoning education, this review aims to contribute to the refinement of educational strategies and assessment methods used in academic and continuing education programs. The insights gained from this review will help educators develop more effective semantic approaches for teaching or learning clinical reasoning, as opposed to fragmented, purely symptom-based or probabilistic approaches. Besides, the results may suggest some ways to address challenges related to the assessment of clinical reasoning and ensure that the assessment tasks accurately reflect learners’ developing competencies and educational progress.

**International Registered Report Identifier (IRRID):**

DERR1-10.2196/50797

## Introduction

### Overview

Health care professional education has made significant progress in teaching clinical reasoning, a fundamental competency for professional practice. Clinical reasoning encompasses the cognitive and metacognitive processes necessary for clinical decision-making [1,2]. These processes enable health care professionals to understand the meaning of clinical data, make accurate decisions, and develop appropriate treatment plans [3,4]. Clinical reasoning is deeply intertwined with the context and environment in which it occurs rather than being disembodied from clinical situations [5].

Insufficiently developed clinical reasoning poses a risk of incidents or errors, which can negatively impact the quality and safety of care [6-8]. Therefore, promoting the development of clinical reasoning in both education and practice is crucial to ensure safe and effective care [2]. In that sense, many efforts have been made to develop different educational strategies aiming to foster the development of clinical reasoning (eg, case studies, concept mapping, simulation, and serious games) [9] and to assess competency (eg, scoring rubrics used during an objective structured clinical examination [OSCE] or a think-aloud exercise) [10]. However, the complexity lies in understanding how these educational strategies and assessment methods can stimulate or simulate the complex cognitive operations involved.

One such cognitive operation is transforming and articulating clinical data, where “raw” data from clinical situations are analyzed, transformed, and expressed using specific professional vocabulary [11]. Translating what students or health professionals hear, see, or perceive into specific professional vocabulary is essential for them to recognize the salient features of a situation and make connections between clinical data and professional knowledge [11]. This cognitive operation is vital in ensuring the consistency and efficiency of oral or written information exchanges between professionals, thus contributing to safe practice [12].

Despite its significance, there are still unanswered questions regarding effective educational strategies to promote the transformation and articulation of clinical data while teaching and learning clinical reasoning. While the analysis of the discourse of students or health care professionals can have a didactic value to promote learning or teaching of clinical reasoning and its assessment, the methods used to achieve this are less clear. In other words, what are the conceptual and methodological tools for analyzing the quality of health care professionals’ discourse? How can we ensure that educational choices are anchored in recognized theories of clinical reasoning to promote the development of professional knowledge structures? How other theories or approaches, particularly on language and health care professionals’ discourse, can support the learning or teaching and assessment of clinical reasoning?

Additionally, understanding how the cognitive process of transformation and articulation of clinical data can be analyzed and assessed is crucial, particularly considering the rapid growth of natural language processing (NLP) in artificial intelligence. NLP aims to develop machines capable of modeling and reproducing human language capabilities [13-16]. Applications of NLP include language translation aids, chatbots (eg, ChatGPT [OpenAI]), and voice-controlled technologies [13]. In clinical practice, for example, NLP can extract relevant information, categorize data, and identify patterns from large volumes of text in electronic health records. In education, NLP enables realistic interactions between students and simulated patients in training software.

Furthermore, challenges persist for educators’ assessment of clinical reasoning, in an educational context where machines attempt to replicate competencies such as clinical reasoning. These could support learning or, on other occasions, present threats to the validity or veracity of students’ cognitive efforts in the assigned tasks assigned to them. [17]. Assessment-related challenges include operationalizing the necessary validity and reliability standards and ensuring that the assessment tasks accurately reflect learners’ developing competencies and educational progress.

Thus, understanding how the teaching and learning of clinical reasoning align with learners’ NLP, specifically the transformation and articulation of clinical data, becomes crucial. Therefore, this scoping review protocol aims to map educational strategies to promote the transformation and articulation of clinical data among students and health care professionals. A secondary objective is to explore the methods used to assess these individuals’ transformation and articulation of clinical data.

### Background

Research addressing the quality of language and discourse in clinical reasoning grew in the 1990s when George Bordage [11,18,19] introduced prototype theory. According to this cognitivist theory, the characteristics of clinical cases (eg, signs and symptoms, diagnosis, and treatment) are not directly stored in long-term memory. Instead, these characteristics undergo a process of abstraction, generating a set of generic characteristics or a brief, typical description of the case known as a prototype.

Prototypes play a significant role in health education as they facilitate creating and consolidating unit-meaning knowledge networks in long-term memory [12]. This allows students and health care professionals to decode the characteristics of a problem. These characteristics will be correlated or contrasted with those from other clinical problems in similar and different contexts [20], leading to densifying the scripts of students and health professionals, thus promoting the development of clinical reasoning. Professional knowledge and in-depth understanding of these prototypes in terms of pathophysiology, concomitant factors, evolution, and so forth, are therefore essential to the clinical reasoning process [21]. In line with prototype theory, the theory of scripts postulates that well-organized knowledge networks and units of meaning stored in the memory of students and health professionals enable effective clinical reasoning [2,22]. Continuing, deliberate clinical practice allows them to densify their scripts by encountering diverse patients and cases.

It was through the use of conceptual and methodological tools derived from structural semantics that Lemieux and Bordage [20,23] analyzed the quality of clinical reasoning, in particular the capacity to diagnose. Issued from linguistics and semiotics (the study of signs and symbols in language), the object of structural semantics is to study the systems of meaning comprised in discourse [20,23]. According to medical semiotics, signs and symptoms represent 2 constituting units of clinical reasoning, highlighting that they must be interpreted in their contexts [24,25]. In structural semantic analysis, the discourse is therefore examined according to the linear and vertical dimensions. The linear dimension relates to the syntactic order, before and after, of the terms used (eg, signs and symptoms) [20,23]. The vertical dimension refers to the underlying semantic structures that allow a student or a health care professional to classify the meaning of clinical data into multiple levels of signification called semantic axes. The semantic axes represent logical levels of abstractions where the student or health professional uses qualitative semantic properties, which are structured on an axis into opposition pairs. Opposition pairs refer to various concepts, such as temporality (constant or intermittent and sudden or gradual), body space (left or right and proximal or distal), degree of impairment (low, moderate, or high), and the quality of the signs and symptoms (apparent or insidious) [20,23].

According to their approach, it is from the constituent units (signs and symptoms) and their forms (eg, syndrome, affected systems, processes, and predisposing factors) that the clinical discourse of students and health professionals can be analyzed, integrating cognitive operations in the latter (eg, being able to decode or define signs and symptoms, to classify them or associate them with others, and to prioritize data). Analyzing the students’ and health professionals’ discourse provides insight into their knowledge’s richness and organization. The semantic component of discourse involves semantic qualifiers, which reflect the intelligible organization of knowledge into meaning units [11,18]. These semantic qualifiers, such as “acute” versus “chronic” pain or “proximal” versus “distal” region, serve as building blocks for organizing knowledge and function as cue mechanisms in long-term memory. Qualifiers can be seen as “useful adjectives” that characterize clinical data abstraction in a situation [12,26].

The syntactic component of discourse is linked to its richness and structure, as it mirrors the richness and organization of one’s knowledge repertoire [11,18]. It refers to the associative competence between the constituent units (signs and symptoms) of the discourse as opposed to a simplistic enumeration of signs and symptoms without creating meaning or relationships between them [20,23]. In this sense, discourse can also appear scattered or reduced when there is an apparent lack of or insufficiently developed knowledge to understand and respond effectively to a situation*.* Bordage [11,18] proposed a discourse classification system that examines the organization of knowledge and the ability to contrast diagnostic hypotheses using clinical data. These categories are (1) reduced: discourse that lacks any effort of semantic transformation, with no connections between patient data and knowledge; (2) scattered: discourse that exhibits limited semantic transformation and disordered hypotheses, which do not reference the obtained data and are listed without contrasting; (3) elaborated: discourse with numerous semantic transformations used judiciously to contrast hypotheses; and (4) compiled: the individual immediately recognizes a semantic data set associated with a clinical hypothesis.

The semantic and syntactic components of discourse are important because they reflect the elaboration and organization of clinical knowledge in the minds of students and health care professionals [11,18]. Semantic qualifiers, integral components of professional vocabulary, are crucial in clearly communicating a patient’s condition in the clinical setting. By mastering these qualifiers, students can effectively convey relevant clinical information and enhance their ability to participate in meaningful clinical discussions [12].

While the semantic competence of students and health professionals should be enhanced to understand, analyze, and develop or evaluate clinical reasoning, the question is how it is solicited or simulated in the educational strategies and evaluation methods used in health education.

## Methods

### Overview

This scoping review will be based on the methods described by Arksey and O’Malley [27]. A scoping review is a knowledge synthesis method to address exploratory research questions by mapping key concepts, types of evidence, and gaps in a research area. This scoping review will identify the extent and range of evidence [28-30] regarding the articulation and transformation of clinical data to guide educational practices and propose avenues of research.

The scoping review will follow the 9-step framework proposed by Peters et al [28] of the Joanna Briggs Institute. This framework includes several consecutive steps that are (1) developing and registering the protocol, (2) formulating the objective and research questions, (3) developing inclusion criteria, (4) identifying relevant studies, (5) selecting relevant studies, (6) charting the data, (7) analyzing the data, (8) reporting the results, and (9) summarizing the results. We will use the PRISMA-ScR (Preferred Reporting Items for Systematic Reviews and Meta-Analyses Extension of Scoping Reviews) checklist [31] to report findings.

### Developing and Registering the Protocol

A preliminary search was performed to assess the existing literature and ensure that no other reviews with the same focus were published. We then developed a protocol based on the 9-step framework mentioned above [28]. The protocol was registered on the Open Science Framework. Furthermore, publishing the protocol in a peer-reviewed journal aims to increase transparency [31].

### Formulating the Objective and Research Questions

In this phase, the 2 primary tasks that are undertaken are (1) identifying research questions that provide a roadmap for the subsequent steps and (2) establishing the scope of inquiry, which encompasses defining the concept, the target population, and the context. The objectives of this scoping review are (1) to map the educational strategies to promote the transformation and articulation of clinical data by students and health professionals and (2) to examine the methods to assess the transformation and articulation of clinical data by students and health care professionals. We have developed the following research questions: (1) what educational strategies are used to promote the transformation and articulation of clinical data among students and health care professionals? and (2) what methods are used to assess the articulation and transformation of clinical data by students and health professionals?

### Developing Inclusion Criteria

Peters et al [28] recommended that inclusion criteria are based on the Population-Concept-Context framework instead of the Population-Intervention-Comparator-Outcomes framework suggested in the PRISMA-P (Preferred Reporting Items for Systematic Review and Meta-Analysis Protocols) [32]. Textbox 1 summarizes the inclusion and exclusion criteria.

**Inclusion and exclusion criteria.**

**Inclusion criteria**

- Studies on students and health professionals
- Studies reporting educational or assessment strategies involving semantic transformation and articulation of clinical data
- Studies written in French or English
- All primary studies, regardless of study design and geographic areas
- Studies conducted in both academic and clinical settings across all geographic areas

**Exclusion criteria**

- Studies in fields other than health education programs, unlicensed health care providers, personal care providers, and caregivers
- Studies without a description of educational or assessment strategies; studies focusing on the developments and applications of intelligent or clinical decision support systems
- Studies in languages other than English or French
- Conference abstracts, protocols, editorials, expert opinions, commentaries, letters, book reviews, blogs and social media

For the population, we will consider literature that discusses educational strategies to foster the development of clinical reasoning among students and health professionals across various academic levels (eg, pregraduates and postgraduates) and disciplines. The included studies will encompass a range of health disciplines, such as medicine, midwifery, occupational therapy, speech-language therapy, and nursing. Conversely, we will exclude studies involving populations outside the field of health, unlicensed health care providers, personal care providers, and caregivers.

The central concept of this review will be the semantic transformation and articulation of clinical data during clinical reasoning [11]. This cognitive operation involves converting “raw” data from a clinical situation into specific professional vocabulary to describe the situation [11]. By organizing clinical data into meaningful units, this cognitive operation helps to understand the situation and generate clinical hypotheses. Clinical reasoning is a cognitive process underlying clinical judgment and decision-making [1,2,33]. These terms, often used interchangeably in the literature [1,4], will also be considered in the search strategy.

For the context, we will consider sources reporting on educational strategies or assessment methods involving the semantic transformation and articulation of clinical data in the context of clinical reasoning. These educational strategies may include, for example, case studies, concept mapping, and simulation. The assessment methods refer to the scoring grid used during OSCE or a think-aloud exercise, and so forth [10]. This review will consider studies conducted in both academic and clinical settings across all geographic areas. Studies examining the developments and applications of intelligent or clinical decision-support systems will be excluded, as technical or engineering procedures could be difficult to analyze and would deviate from the objectives of the review.

We will consider primary studies with quasi-experimental (eg, before and after studies and interrupted time series), experimental (eg, randomized controlled trials), observational (eg, cohorts, case-control, and cross-sectional), qualitative, and mixed methods designs. We will also include grey literature such as theses, dissertations, conference proceedings, and research reports. To focus on scientific literature with sufficient content to help answer our questions, the following will be excluded: conference abstracts, protocols, editorials, expert opinions, commentaries, letters, book reviews, blogs, and social media. Studies written in French or English will be included. The search will span the period from 1990 to the present, that is, after Bordage introduced semantic qualifiers in the 1990s. The objective is to comprehensively examine the literature on clinical reasoning in health professional education during this timeframe.

### Identifying Relevant Studies

As recommended by Peters et al [28], a comprehensive 3-step search strategy was implemented with the support of a health science librarian. Initially, an exploratory search was performed in CINAHL and PubMed to identify keywords and medical subject headings from articles about the review topic. Subsequently, a thorough literature search was conducted in November 2022 using 5 databases: CINAHL (EBSCOhost), MEDLINE (Ovid), Embase (Ovid), PsycINFO (Ovid), and Web of Science (Clarivate). The search strategy was first developed in CINAHL and later adapted to other databases (see Multimedia Appendix 1). Considering the possibility of articles being present in fields other than health care, such as education or linguistics, the review team decided to manually select articles that specifically focused on students or health professionals to ensure relevance to the research topic.

In the future, we plan to use a hand search approach to review the reference list of the included records. This approach will help identify any additional relevant studies that may have been missed during the initial database search. Furthermore, we intend to search for unpublished studies using the ProQuest dissertations and theses electronic database and the ProQuest TDM studio tool and conduct targeted searches on Google Scholar. For the Google Scholar searches, we will use specific search string queries such as “semantic transformation and clinical reasoning,” “diagnostic reasoning and semantics,” and “semantic qualifiers and differential diagnosis.” We will analyze Google Scholar’s top 100 search results to identify additional studies aligning with the review questions and objectives.

### Selecting Relevant Studies and Charting the Data

After conducting the initial database search, the retrieved references were imported into Covidence (Veritas Health Innovation) to facilitate the screening process and identify duplicate records. To ensure consistency and establish a shared understanding, 3 reviewers independently assessed the eligibility of a randomly selected sample of 25 articles based on the predefined inclusion and exclusion criteria. In all, 2 meetings were conducted to refine the selection criteria to improve agreement rates.

The screening of titles, abstracts, and full texts was carried out independently by 2 reviewers, following the predefined selection criteria. In the event of any disagreements, a third reviewer was involved. The reasons for excluding studies at the full-text stage were documented and will be reported accordingly.

In the upcoming phase of the scoping review, 2 independent reviewers will extract relevant data from all included studies. A structured data extraction form developed by the reviewers will be used to record the information. Microsoft Excel will be used for data management. The extraction form proposed by Peters et al [28] will serve as a foundation, encompassing article characteristics such as the first author’s name, year of publication, and country of origin, as well as study methods, including the aim, study design, and population. Additionally, we will create codes under the following categories: the transformation and articulation of clinical data to facilitate learning or capture clinical reasoning; educational strategies; any referenced theoretical framework; methods or tools used to analyze the articulation and transformation of clinical data; relevant results; and authors’ recommendations.

Following the recommendation of Peters et al [28], the extraction form will undergo a testing phase by the reviewers, who will extract data from 5 selected studies. This process will help ensure the effectiveness and consistency of the form. Based on their feedback, the reviewers will collaboratively refine the extraction form and create a comprehensive list of codes if any adjustments or modifications are required.

Subsequently, 1 reviewer will perform data extraction, and a second reviewer will independently review the results to ensure accuracy and reliability. In the event of any discrepancies, the reviewers who performed the data extraction will engage in discussion to reach a consensus. A third reviewer will be consulted to finalize the extracted data if a consensus cannot be reached.

### Analyzing the Data and Reporting and Summarizing the Results

Extracted data will be processed using content analysis techniques inspired by Miles et al [34]. This data analysis method involves three steps, which are (1) data condensation, (2) data display of similarities and differences, and (3) drawing and verifying conclusions. Based on the review questions, relevant findings pertaining to the characteristics of the included studies will be presented in tables, along with insights into the transformation and articulation of clinical data for educational and assessment purposes. In addition, a narrative report will be used to summarize the key findings from the articles. For this purpose, a brainstorming session involving all reviewers will be organized. The findings will be described with respect to the research questions and the objectives of this scoping review. Additionally, gaps or limitations in the existing literature will be identified and highlighted to provide insights for future research. The search results and the study inclusion process will be reported comprehensively in the final report using a PRISMA-ScR flow diagram [31].

### Ethical Considerations

All data will be collected from published and grey literature. Ethics approval is, therefore, not a requirement. We will present our findings at relevant conferences and submit them for publication in peer-reviewed journals.

## Results

**Figure 1:**
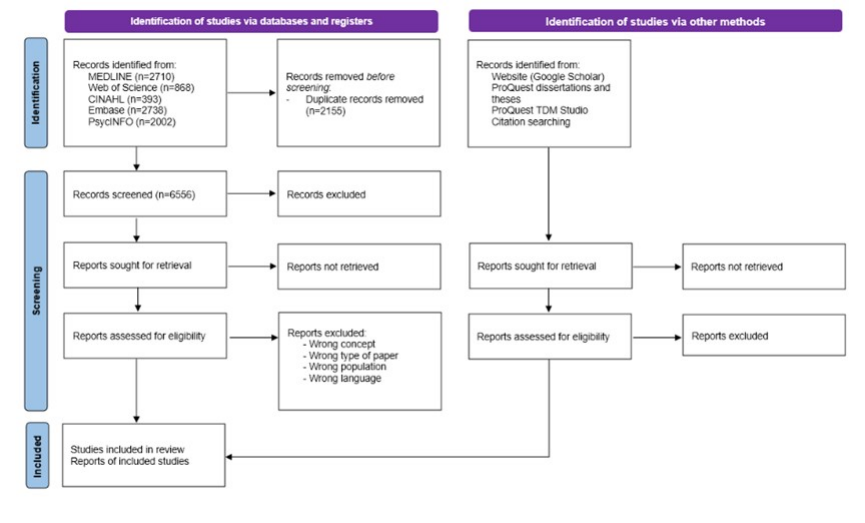
PRISMA (Preferred Reporting Items for Systematic Reviews and Meta-Analyses) flow diagram.

Database searches were conducted in November 2022. A total of 8711 results were retrieved, of which 2155 were duplicates. Figure 1 shows a flow diagram of the records identified. The review will be completed by February 2024.

## Discussion

### Principal Findings

The proposed scoping review aims to provide a comprehensive overview of the existing evidence on semantic transformation and articulation of clinical data during clinical reasoning education. The initial literature search yielded substantial results, totaling 6656 after removing duplicates.

Various educational strategies have been identified thus far. These include summary statements with simulated patients, verbal analysis of thoughts related to vignettes, tests to assess the association between clinical data and diagnoses, and examining written reports in actual or simulated contexts. Furthermore, the review has uncovered the presence of rubrics that incorporate both the semantic and syntactic dimensions of students’ and health professionals’ discourse, providing a framework for evaluating clinical reasoning. These findings highlight the diversity of educational strategies and assessment tools used in the field. Based on the review’s preliminary findings, an initial evaluation of the theories about the articulation and transformation of clinical data has been formulated.

Despite a robust review design, some limitations can be identified in this protocol. This scoping review may exclude relevant studies as it will mainly include peer-reviewed journal articles. As such, some relevant grey literature may be excluded from our findings. Excluded studies examining developments and applications of intelligent or clinical decision support systems could contain some additional information to answer the review questions. Indeed, the keywords used in the search strategy are broad and may not identify all specialized studies. Moreover, only considering English and French as the languages of publication could exclude papers relevant to our scoping review written in other languages.

### Conclusions

By synthesizing the evidence on semantic transformation and articulation of clinical data during clinical reasoning education, this review aims to contribute to the refinement of educational strategies and assessment methods used in academic and continuing education programs. The insights gained from this review will help educators develop more effective semantic approaches for teaching or learning clinical reasoning, as opposed to fragmented, purely symptom-based or probabilistic approaches. Besides, the results may suggest some ways to address challenges related to the assessment of clinical reasoning and ensure that the assessment tasks accurately reflect learners’ developing competencies and educational progress.

## Appendix

Multimedia Appendix 1 Search strategy.

## Abbreviations

NLP: natural language processing
OSCE: objective structured clinical examination
PRISMA-P: Preferred reporting items for Systematic Review and Meta-Analysis Protocols
PRISMA-ScR: Preferred Reporting Items for Systematic Reviews and Meta-Analyses Extension for Scoping Reviews

## Data Availability

The data sets generated and analyzed during this study are available from the corresponding author on reasonable request.
